# Case report: Prompt response to radiotherapy and chemotherapy combined with crizotinib in gingival sarcomatoid squamous cell carcinoma with MET 14 mutation

**DOI:** 10.3389/fonc.2022.1006516

**Published:** 2022-09-06

**Authors:** Zhenhua Sun, Bingjie Xia, Ming Zhang, Shuai Xu, Yingqian Ma, Xianbo Zhang

**Affiliations:** ^1^ Department of Radiation Oncology, Hebei General Hospital, Shijiazhuang, China; ^2^ Graduate School of North China University of Science and Technology, Tangshan, China; ^3^ Graduate School of Hebei North University, Zhangjiakou, China

**Keywords:** gingival carcinoma, MET, crizotinib, radiotherapy, chemotherapy

## Abstract

**Background:**

As a kind of squamous cell carcinoma of head and neck (HNSCC), gingival sarcomatoid squamous cell carcinoma (GSSCC) is a rare biphasic malignant neoplasm. To date, surgical resection was often utilized for gingival squamous cell carcinoma (GSCC), while for patients with advanced gingival carcinoma who cannot tolerate surgery, radiotherapy and chemotherapy can be regarded as a treatment strategy. Many molecular-targeted drugs were investigated and approved for the treatment of malignant diseases, including hematologic diseases and solid tumors. Although targeted therapies such as EGFR inhibitors have shown therapeutic efficacy in HNSCC, there are still some patients who cannot benefit from it. New therapeutic targets and strategies should be further explored.

**Case presentation:**

An 83-year-old woman was referred to our hospital with left lower gingival mass for more than 1 month in June 2021. Pathologic diagnosis is sarcomatoid squamous cell carcinoma. Due to the large tumor at the time of diagnosis and poor quality of life, the patient was intolerant to surgery, so she was given radiotherapy (RT) combined with concurrent chemotherapy (CT) with albumin bound paclitaxel. According to next-generation sequencing (NGS) results (MET exon 14 skipping mutation-positive), she was treated with crizotinib, a tyrosine kinase inhibitor that targets MET. Through the comprehensive treatment, the patient’s condition promptly improved, clinical complete remission (CR) was achieved in 2 months, and 9-month progression-free survival (PFS) was obtained. She finally died from non-cancer-related diseases.

**Conclusion:**

Here we report the treatment of a GSSCC patient with MET mutation, who responded to crizotinib promptly and positively. It provides a new reference for understanding MET abnormalities in GSSCC and offers a new idea for the targeted treatment of gingival carcinoma.

## Introduction

Gingival carcinoma is a relatively rare malignancy that accounts for 10% of all oral cancers in Europe and the United States. Gingival carcinoma has the second highest incidence of oral cancer after tongue carcinoma. Its common pathological type is squamous cell carcinoma (SCC), while sarcomatoid squamous cell carcinoma is very rare (1, 2). The main therapy for gingival carcinoma is surgery alone or a comprehensive treatment based on surgery. However, early GSCC diagnosis remains challenging. Roughly 70% of patients have inoperable or metastatic disease at diagnosis. According to data, cumulative 5-year survival after the diagnosis was 43%, while at 10 years it was 11% (3). Furthermore, surgery can cause not only a disfiguring and poor quality of life but also financial and psychological burden for patients. Concurrent chemoradiotherapy (CRT) is an important treatment for locally advanced HNSCC patients, which can well-preserve organ and function integrity and improve the local control rate. Targeted therapy for gingival carcinoma is still in the exploratory stage. This case report is the first reported case of a patient with advanced gingival squamous cell carcinoma sarcomatoid lesions with MET gene mutation that achieved clinical complete remission after treatment with crizotinib targeted therapy combined with CRT.

## Case presentation

**Figure 1:**
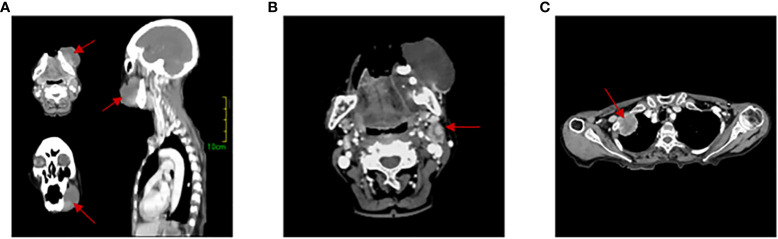
Computed tomography (CT) scans. **(A)** 3D image of gingival tumor. **(B)** Lymph node metastasis area. **(C)** Lung metastases lesion. The red arrow indicates the location of the lesion.

**Figure 2:**
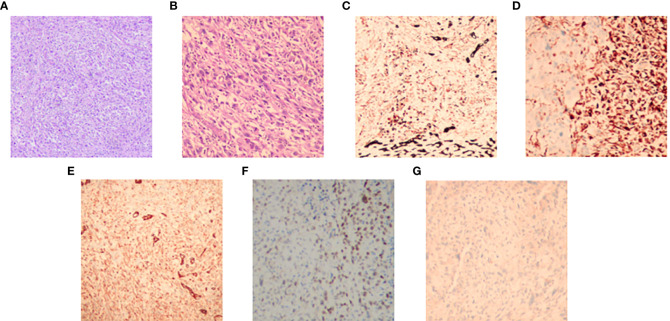
The hematoxylin–eosin (H&E) and immunohistochemical pictures of the tumor. **(A)** H&E, original magnification, ×100. **(B)** H&E, original magnification, ×400. Immunohistochemistry: CKpan **(C)**, Vementin **(D)**, EMA **(E)**, P63 **(F)**, and S100 **(G)**.

An 83-year-old woman was referred to our hospital with left lower gingival mass for more than 1 month in June 2021. The patient presented with progressive enlargement of gingival swelling and discomfort such as swollen and painful gums and difficulty in opening the mouth for 1 month. The patient was first diagnosed in our hospital, with a healthy past and no special family history. Computed tomography (CT) scan revealed an oval-shaped mass in the left lower gingival area with adjacent mandibular bone destruction and multiple enlarged lymph nodes in the neck and left submandibular area, and an occupying lesion in the upper lobe of the right lung (**Figure 1**). PET/CT scan showed no other metastases. Physical examination revealed a mass of about 6 cm located in the left lower gingival area, causing difficulty in opening the mouth. Multiple enlarged lymph nodes were palpable in the left neck. According to laboratory testing, red blood cell count was 2.77 × 10^12^/L, hemoglobin 77 g/L, albumin 32.2 g/L, and the results of the biomarkers CA153, CEA, CA199, CA125, and SCC showed no abnormalities. Histopathological examination revealed an actively growing spindle cell neoplasm, which tends to be a poorly differentiated sarcomatoid variant of squamous cell carcinoma. The pathology of the right pulmonary nodule was confirmed to be metastasis. Immunohistochemistry (**Figure 2**) outcomes were presented as follows: AE1/AE3 (+), P63 (+), EMA (+), CKpan (+), Vimentin (+), Ki67 (80%), EGFR (70%), S100 (-), CK5/6 (-), P40 (-), CK7 (-), and PD-L1 (DAKO 22C3) (CPS: 50). The patient was clinically diagnosed with cT4aN2bM1 stage IVB gingival carcinoma (according to the AJCC).

**Figure 3:**
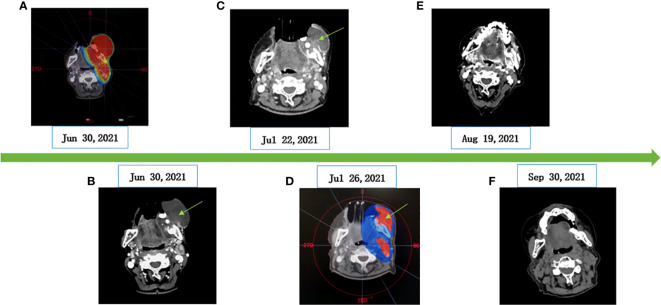
CT imaging of changes of gingival masses. **(A)** RT dose distribution image. **(B)** Before CRT (62 mm×53 mm×64 mm). **(C)** CRT and crizotinib for 1 week (50 mm×32 mm×42 mm). **(D)** Adjust the radiotherapy area (gingival mass: 8mm×27mm×46mm). **(E)** CRT and crizotinib for 1 month (undetectable tumor size). **(F)** Oral crizotinib for 2 months (efficacy evaluation as CR).

The patient poorly tolerated and refused surgery and chose CRT as the primary treatment. The patient started intensity modulated radiation therapy (IMRT) along with albumin-bound paclitaxel. Concurrent chemoradiotherapy began on 5 July 2021 and ended on 18 August 2021. The details of the regimen are as follows. Radiotherapy was given five times a week (from Monday to Friday). Radiotherapy planning: PGTV (gingival mass and metastatic lymph node) 66Gy/33F/7W, 2Gy/F; PGTV2 (lung metastases lesion): 66Gy/33F/7W 2Gy/F; PTV (lymphatic drainage area): 59.4Gy/33F/7W 1.8Gy/F. Chemotherapy regimen: albumin-bound paclitaxel (200 mg/m^2^, d1, Q3W), a total of two cycles of single-agent chemotherapy. In order to search for targetable therapeutic drugs, we performed NGS on the patient. The NGS of the gingival biopsy specimen indicated a MET exon 14 skipping mutation (c.3028+3A>G), with an abundance of 49.67%, which can potentially cause alternative splicing of the MET protein. There was no significant change in the tumor volume during the first week of CRT. According to the results of the NGS, the patient was treated with crizotinib (250 mg, po qd) on 15 July 2021. The tumor volume decreased significantly after 1 week of crizotinib combined with CRT. During the treatment, two times of tissue cleanings were given due to the large amount of tumor necrosis tissue, and the target area was redrawn after each cleaning. In order to protect the surrounding normal tissues and avoid toxic reaction such as radiation-induced mandibular inflammation, CT simulation and target delineation of radiotherapy was re-performed on 26 July after cleaning up the necrotic tissues. The plan was reevaluation to ensure a high dose in the tumor site (**Figure 3**). This patient responded positively to crizotinib-targeted therapy, and no serious adverse effects of CRT were observed during treatment. Chemotherapy was only synchronized with radiotherapy for two cycles. After the end of CRT, the patient refused to continue chemotherapy and received oral crizotinib treatment at home. Two months later, reevaluation by CT scans showed complete remission (CR) in the gingival area. Followed up by telephone, the patient took crizotinib orally for 8 months, after which it was interrupted due to financial reasons. One month later, she died of pneumonia caused by a cold. The infection has been ruled out to be related to radiation pneumonitis and crizotinib treatment.

## Discussion

Sarcomatoid squamous cell carcinoma (SSCC), also known as spindle cell squamous cell carcinoma, is a rare and peculiar biphasic malignant neoplasm that occurs mainly in the larynx, but also in other mucosal areas such as the gingiva, tongue, pharynx, and nasal cavity, accounting for 0.4%–4% of HNSCC (4). GSCC accounts for less than 1% of all tumors in the oral region, and GSSCC is even rarer. In the existing reports, SSCC is more difficult to treat than SCC, and the prognosis is worse (5). The National Comprehensive Cancer Network Guidelines (NCCN) and previous reports recommend surgery as the primary treatment for GSCC. Moreover, radical neck dissection is the standard treatment for metastatic lymph nodes. For advanced GSCC patients who decline or cannot tolerate surgery, concurrent or sequential chemoradiotherapy is an alternative (6, 7).

With the development of precision medicine, the activation of the epidermal growth factor receptor (EGFR) is observed in HNSCC. The EGFR becomes one of the promising molecular targets for treating solid tumors including it. Cetuximab is an EGFR-targeted monoclonal antibody that improved the OS of HNSCC patients in both locally advanced and recurrent/metastatic settings, which was approved to be used as the standard treatment option in in the USA and Japan (8). The overexpression of the vascular endothelial growth factor (VEGF) in HNSCC also attracts people’s attention, while most studies did not show clinical benefit of angiogenesis inhibitors such as bevacizumab, tyrosine kinase inhibitors, and endostatin (9).

The MET proto-oncogene encodes for receptor kinase c-Met and binds to MET’s ligand hepatocyte growth factor. Binding of the ligand to the receptor induces dimerization and activates the receptor, and recruits proteins involved in signal transduction like Grb2, Shc, Src, and phosphoinositide 3’ kinase. This, in turn, leads to downstream cascades in pathways involved in cell apoptosis and cellular motility and invasion. Aberrant MET signaling drives the tumor growth through increased cell proliferation, survival, invasion, and metastasis (10, 11). Abnormal activation of MET involves MET exon 14 skipping mutation, MET amplification, and MET protein overexpression. Point mutations, deletions, and insertions might lead to exon 14 skipping mutation (12).

Several small-molecule TKI inhibitors targeting MET are being evaluated for the treatment of patients who have MET exon 14 skipping mutations. These drugs include nonselective type 1a inhibitors (e.g., crizotinib) and selective type 1b inhibitors (e.g., tepotinib, savolitinib, and capmatinib). Tepotinib has durable antitumor activity in patients with advanced NSCLC with MET exon 14 skipping mutations (13). Savolitinib yielded promising activity and had an acceptable safety profile in patients with pulmonary sarcomatoid carcinoma and other NSCLC subtypes positive for METex14 skipping alterations (14). Crizotinib is approved for the treatment of ALK- or ROS1-rearranged advanced NSCLCs. Apart from its activity against ALK and ROS1, it has potent activity against MET and low nanomolar potency in cell lines that harbor MET exon 14 alterations (15). A study showed that crizotinib is active in advanced NSCLC patients with MET exon 14-altered (16). According to the literatures of the past 10 years (**Table 1**), the excellent response to crizotinib in some rare tumor with MET mutation provides a basis for treatment of this patient.

**Table 1:** The excellent response to crizotinib in some rare tumor with MET mutation.

Case	Gender	Age(years)	Tumor	MET mutation	OP	CT	RT	Achieve PR/CR(months)
**1 (** 17)	M	53	Tongue Squamous-Cell carcinoma	exon 14-altered	YES	YES	YES	CR,1
**2 (** 18)	F	41	Intrahepatic cholangiocarcinoma	EHBP1-MET Fusion	NO	NO	NO	PR,1
**3 (** 19)	F	66	Sinonasal undifferentiated carcinoma	amplification	YES	YES	YES	PR,1
**4 (** 20)	M	51	papillary renal carcinoma	amplification	YES	NO	YES	PR,3
**5 (** 21)	F	19	Histiocytic sarcoma	exon 14-altered	NO	YES	YES	PR,1
**6 (** 22)	M	51	Gastric cancer	amplification	NO	YES	NO	CR,2
**7 (** 23)	F	59	Carcinoma of unknown primary	amplification	NO	YES	NO	PR,7
**8 (** 24)	F	55	Gallbladder cancer	amplification	YES	YES	NO	PR,2

Op, operation; CT, chemotherapy; RT, radiotherapy; PR, partial remission; CR, complete remission.

In this case, the elderly patient with an advanced sarcomatoid variant of gingival squamous cell carcinoma and lung metastases was intolerant to surgery. Considering the rare tumor pathological type, NGS of the tumor tissue was performed. The result was unexpected, which revealed the presence of oncogenic mutation c.3028+3A>G in the MET gene, exon 14. Therefore, we combined crizotinib targeted therapy with concurrent chemoradiotherapy. After crizotinib treatment, her clinical symptoms promptly improved without obvious adverse reactions.

At the first visit, the patient had a high tumor load, which seriously affected oral intake. An Eastern Cooperative Oncology Group performance status of 3, a Nutrition Risk Screening 2002 score of 5, and a body mass index of 17.5 kg/m^2^ indicated nutritional risk. During the treatment, we administered enteral nutritional support to the patient with nasal feeding tubes and ensured oral hygiene during radiotherapy to avoid secondary infection caused by necrotic tissue. The patient’s nutrition index improved significantly that she could tolerate chemoradiation. There was significant tumor shrinkage and necrosis through the course of the treatment. The patient needed assurances about the effectiveness and remission of the treatment at this point. To avoid affecting the biological dose effect in the target area and protecting normal tissues as much as possible due to lack of oxygen and the occupying effect of necrotic tissues, we closely observed the changes of tumor tissues during the treatment, cleaned up the necrotic tissues in time, and repositioned and outlined the target area to make the plan, thus ensuring the treatment effect.

The patient reached CR in 2 months through comprehensive treatment. This case provides strong evidence for the application of crizotinib-targeted therapy for sarcomatoid lesions of advanced gingival squamous cell carcinoma with MET gene mutations, and provides new diagnostic ideas and inspiration for the comprehensive treatment of advanced gingival cancer. For patients with clinically rare tumors and large tumor loads and elderly patients who cannot tolerate surgery and whose surgery may lead to aesthetic impairment, precision testing to find therapeutic targets combined with simultaneous radiotherapy and chemotherapy and other comprehensive treatments may provide patients with improved quality of life and survival benefits.

## Data availability statement

The original contributions presented in the study are included in the article/Supplementary Material. Further inquiries can be directed to the corresponding author.

## Ethics statement

Written informed consent was obtained from the individual(s) for the publication of any potentially identifiable images or data included in this article.

## Author contributions

ZS and BX contributed equally to this work and share first authorship. MZ conceived and designed the research. ZS, BX, SX, YM, and XZ performed the research and/or analyzed data. ZS, BX, and MZ wrote the paper. All authors contributed to the article and approved the submitted version.

## Acknowledgments

The authors are grateful to the patient who participated in this study and also thank colleagues in pathology and radiology of our hospital.

## Conflict of interest

The authors declare that the research was conducted in the absence of any commercial or financial relationships that could be construed as a potential conflict of interest.

## Publisher’s note

All claims expressed in this article are solely those of the authors and do not necessarily represent those of their affiliated organizations, or those of the publisher, the editors and the reviewers. Any product that may be evaluated in this article, or claim that may be made by its manufacturer, is not guaranteed or endorsed by the publisher.
